# Acupuncture on treating asthma: A protocol for systematic review and meta analysis

**DOI:** 10.1097/MD.0000000000018457

**Published:** 2020-01-03

**Authors:** Yan-Ming Chen, Xiao-Lei Xie, Peng-Yun Xiao, Qiu-Hong Wang, Ji-Sheng Wang, Xu-Dong Yu, Sheng Deng

**Affiliations:** a Department of Acupuncture and Moxibustion, Changping District Chinese Medicine Hospital; b Student Office, Capital Medical University; c Department of Respiratory, Shunyi District Chinese Medicine Hospital; d Department of Andrology, Dongzhimen Hospital, Beijing University of Traditional Chinese Medicine, Beijing, China.

**Keywords:** acupuncture, asthma, systematic review

## Abstract

**Background:**

Asthma is one of the most common chronic diseases in the world, with approximately 300 million asthma patients worldwide. The mortality rate of asthma is 1.6 to 36.7 / 100,000 people, and China has become one of the countries with the highest asthma death rate in the world. Asthma is a chronic allergic airway inflammatory disease. Patients with this disease may have symptoms such as cough, wheezing, and difficulty breathing. For many years, Western medicine has mainly used anti-inflammatory, anti-bronchial spasm, asthma, cough and oxygen to treat this disease, but the effect is not good. Clinical studies in recent years have found that the use of acupuncture in the treatment of bronchial asthma has a good clinical application prospect. This study was conducted to study the effect of using acupuncture to treat asthma.

**Methods and analysis:**

We will search for PubMed, Cochrane Library, AMED, EMbase, WorldSciNet; Nature, Science online and China Journal Full-text Database (CNKI), China Biomedical Literature CD-ROM Database (CBM), and related randomized controlled trials included in the China Resources Database. The time is limited from the construction of the library to November 2019. We will use the criteria provided by Cochrane 5.1.0 for quality assessment and risk assessment of the included studies, and use the Revman 5.3 and Stata13.0 software for meta-analysis of the effectiveness, recurrence rate, and symptom scores of asthma.

**Ethics and dissemination:**

This systematic review will evaluate the efficacy and safety of acupuncture for asthma. Because all of the data used in this systematic review and meta-analysis has been published, this review does not require ethical approval. Furthermore, all data will be analyzed anonymously during the review process Trial.

## 1 Introduction

Asthma is also known as bronchial asthma. Bronchial asthma is a pulmonary paroxysmal disease that is more common in the clinic.^[1]^ The disease is mainly caused by sudden exhalation prolongation, difficulty in breathing, wheezing and coughing, irritability, sweating, chest tightness, and other clinical symptoms.^[2,3]^ Among them, the most typical clinical symptom is dyspnea. Bronchial asthma is mainly a chronic inflammatory disease of the airway involving a variety of cells and cellular components such as T lymphocytes, mast cells, airway epithelial cells, eosinophils, and neutrophils.^[4–7]^ Most scholars believe that there is a certain relationship between airway chronic inflammatory diseases and airway hyperresponsiveness. Repeated shortness of breath, cough, and wheezing are caused by reversible airflow limitation, especially in the morning or night.^[8]^ Clinical symptoms from different degrees of patients can be divided into clinical remission, acute exacerbation and chronic duration.^[9]^ The clinical remission period refers to the untreated (or treated), and its signs and clinical symptoms can be relieved or even disappeared.^[10,11]^ The lung function of the patient is in a state before the acute attack, and the maintenance time is more than 3 months.^[12,13]^ Chronic duration refers to clinical symptoms such as chest tightness, wheezing, cough, and shortness of breath appearing every week. According to research, at the current stage, global bronchial asthma is as high as 300 million, while in China, it is about 25 million, with a high incidence.^[14]^ The disease seriously affects the quality of daily life of patients and imposes a huge burden on society and families.^[15]^ Serious respiratory illness may occur if not treated promptly or treated improperly, and severe cases may die as the disease progresses. In modern medicine, the cause of bronchial asthma is due to its chronic inflammatory response, and it should be adhered to the principle of symptomatic treatment and anti-inflammatory and antispasmodic treatment.^[16,17]^ Although it has certain therapeutic effects, it is easy to recurrent and the curative effect is greatly reduced.

The causes of asthma include genetic factors, allergens (indoor allergens, occupational allergens, drugs and food additives), triggering factors (air pollution, smoking, respiratory virus infection, perinatal fetus environment, others).^[18–20]^ Asthma needs to be differentiated from diseases such as cardiogenic asthma, bronchial lung cancer, endotracheal lesions, and allergic lung infiltration.

Acupuncture is a component of traditional Chinese medicine. According to the literature, acupuncture can enhance the immune system function of patients and thus play a role in relieving phlegm.^[21]^ According to a number of clinical practices, acupuncture therapy has a considerable effect, has a high technical content, can inhibit allergic reactions, activate its defense system function, and the patient's bronchial smooth muscle tone is reduced, and finally achieve anti-asthmatic purpose.^[22]^

After preliminary searches and database analysis, we found that the frequency of randomized controlled trials (RCT) of acupuncture for asthma is rising.^[23]^ Previous clinical trials have shown that acupuncture can relieve symptoms and improve the quality of life of patients. These effects persist in people with asthma. However, due to the size of the clinical center and the limited number of samples, the current level of evidence-based medical evidence is still insufficient. Therefore, we hope to evaluate the efficacy and safety of acupuncture in the treatment of asthma through meta-analysis, and provide a sufficient basis for its clinical application.^[24]^

## 2 Methods

This systematic review protocol has been registered on PROSPERO CRD42019137864 (https://www.crd.york.ac.uk/prospero/display_record.php?RecordID=137864). The protocol follows the Cochrane Handbook for Systematic Reviews of Interventions and the Preferred Reporting Items for Systematic Reviews and Meta-Analysis Protocol (PRISMA-P) statement guidelines. We will describe the changes in our full review if needed.

### 2.1 Inclusion criteria for study selection

#### 2.1.1 Types of studies

We will gather all studies of acupuncture on treating asthma: a systematic review and meta-analysis which, no matter whether they have been published or not, base on the method of RCT. The language is limited to Chinese and English. Non - RCTs quasi - RCTs, series of case reports and cross research will be excluded.

#### 2.1.2 Types of participants

It will include adult patients diagnosed with asthma, which means there are no restrictions on regions, countries, races, and sources. Both the patient and the family informed the study and signed a consent form.

#### 2.1.3 Types of interventions

We will adopt acupuncture treatment of asthma as experimental interventions. Considering that the theory of pharmaco-acupuncture and point injection belong to another part of TCM, so they will be considered for exclusion.

*Control interventions.* As for control intervention, a person receiving virtual acupuncture treatment can be used as a placebo control, or as a blank control without receiving any treatment. However, once they receive acupuncture combined drugs or other Chinese medicine, the trial will be rejected.

The following treatment comparisons will be studied:

(1) Acupuncture and no treatment;
(2) Acupuncture and placebo/false acupuncture;
(3) Acupuncture and drug treatment;
(4) Acupuncture and other active therapies;
(5) Acupuncture combined with another active therapy compared to the same treatment alone;

#### 2.1.4 Types of outcome measures

*Primary outcomes.* The main criteria are:

1. Symptoms and signs disappear;
2. No significant wheezing after the event;
3. Stop the drug for 3 months without attack;
4. Film degree exam;
5. Lung ventilation

*Secondary outcomes.* Secondary assessment criteria include Lung sounds and cough disappear. At the same time, close attention should be paid to whether adverse reactions or adverse events occur during the experiment to comprehensively evaluate the clinical efficacy and safety of acupuncture in the treatment of asthma.

#### 2.1.5 Electronic searches

Database Search: Search PubMed, Cochrane, Library, AMED, EMbase, WorldSciNet; Nature Science online and China National Knowledge Infrastructure (CNKI), China Biology Medicine disc (CBMdisc). The temporal interval is limited from the time that the databases created to November 2019, and the combination of keyword and free word retrieval is adopted. The search terms include “acupuncture” and “asthma”. The search term in the Chinese database is the translation of the above word. The complete PubMed search strategy is summarized in Table 1.

**Table 1:**
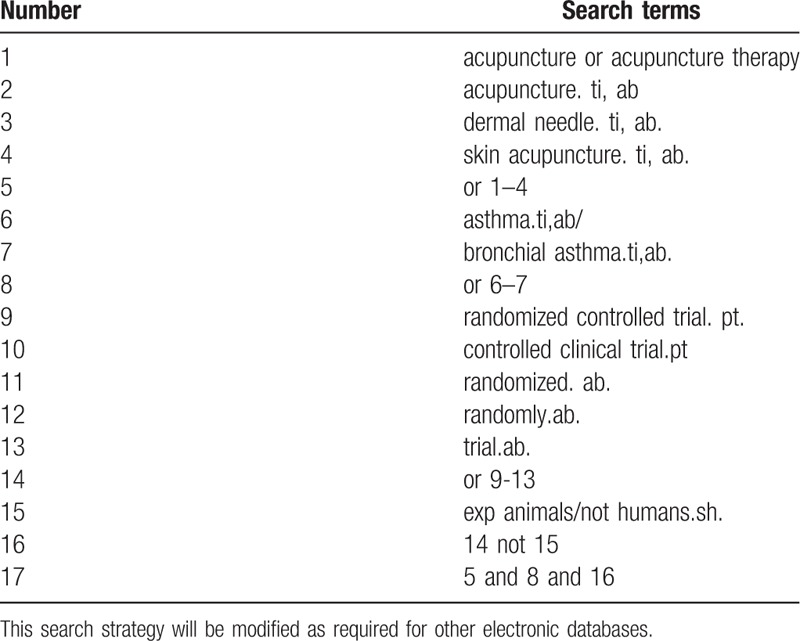
Search strategy used in PubMed database.

#### 2.1.6 Searching other resources

The manual search mainly searched for relevant literatures, earlier than the database above-mentioned, such as “China Rehabilitation Medicine Journal” “Chinese Acupuncture” “Chinese Journal of Physical Medicine and Rehabilitation” “Acupuncture Clinical Journal”, and “Shanghai Acupuncture Journal”.

### 2.2 Data collection and analysis

#### 2.2.1 Study identification

**Figure 1:**
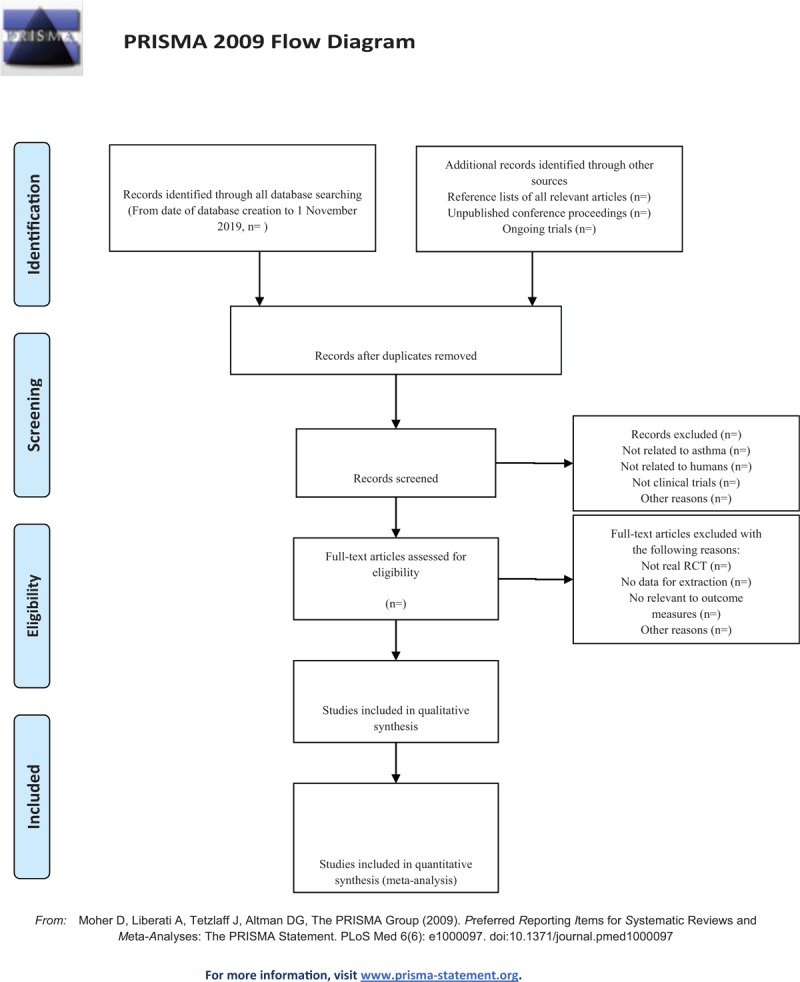
The PRISMA flow chart.

(1) There are 2 researchers filtering out the literature that clearly do not conform to the study such as meeting minutes dissertations reviews animal experiments and so on, which, after excluding all the retrieved documents from the duplicated literature, adopt the method of reading the title of the literature abstracts etc. The details of selection process will be shown in the PRISMA flow chart (Fig. 1).
(2) The second time of screening the literature: Skimming the remaining documents and filtering out unqualified documents such as case reports theoretical discussions and non-conformance of interventions.
(3) The third time of screening the literature: Carefully reading the remaining documents and strictly filtering out unqualified documents such as general controlled trials, lacking control group, deficiency of random allocation, incompatible outcome indicator and the appearance of similar data etc.
(4) As for the literature that cannot be ensured, it would be confirmed by the discussion of the two researchers. And if they cannot reach an agreement, the third-party experts would get involved, which aims at absorbing the appropriate RCTs into the study.

#### 2.2.2 Data extraction and management

The literature data extraction will be completed independently by 2 researchers and the data form uniformly developed by the researcher was filled out. The data extraction content includes the following:

(1) General information: Article title, First author, Corresponding Author, time of publication research, evaluation correspondence, contact information.
(2) Research method: Design pattern, ample size, random allocation, random hiding, blind method, baseline level.
(3) Participants: Patients age, gender, asthma diagnostic criteria, severity, ethnicity study, location.
(4) Intervention: acupuncture, acupuncture point, period of treatment, treatment frequency
(5) Efficacy evaluation: Main observation indicators secondary observation indicators safety indicators and number of adverse reactions.
(6) Note: sources of funds, medical ethics audit, important references

#### 2.2.3 Assessment of risk of bias in included studies

As for the Literature quality evaluation, we will use the bias risk assessment tool recommended by Cochrane to assess the quality of all included literature and risk of bias. The assessment include:

(1) sequence generation;
(2) allocation concealment;
(3) blinding of participants, personnel and outcome assessors;
(4) incomplete outcome data;
(5) selective outcome reporting;
(6) other sources of bias. The evaluation above would be independently evaluated by 2 researchers.

If there are different opinions, we discuss them. If there are still differences exist, we would consult the third appraiser. Otherwise, we need to consult with the Cochrane Professional Group for solution.

#### 2.2.4 Statistical analysis

The meta-analysis studied in this review will adopt Rev Man5.3 and Stata13.0 statistical software. Heterogeneity test will be used for the inclusion of the study, and random or fixed effect models will be adopted, with *P* < .05 as the test standard. If the heterogeneity between the results is too large, the random effects model (REM), which deduce the source of heterogeneity by sensitivity analysis, will be used for the rest analysis. Secondly, according to the different type of statistical data, the binary categorical variable will use the odds ratio (OR) and its 95% confidence interval (CI) as the effect analysis index. As for the continuous variable, the standardized mean difference (SMD) and its 95% CI will be used as the effect analysis index. If the outcome measures only provide the means and standards deviation before or after treatment, the Mean_change_ and the SD_change_ are obtained according to the method provided in Cochrane Handbook 5.1.0: 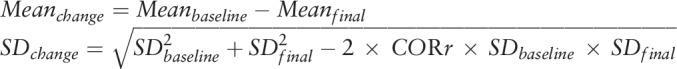


The forest map and funnel plot were drawn and analyzed using Rev Man5.3 software, and the funnel plot was used to analyze potential publication bias. As for the Literature quality evaluation, we will use the bias risk assessment tool recommended by Cochrane to assess the quality of all included literature and risk of bias. The assessment include:

(1) sequence generation;
(2) allocation concealment;
(3) blinding of participants, personnel and outcome assessors;
(4) incomplete outcome data;
(5) selective outcome reporting;
(6) other sources of bias.

The evaluation above would be independently evaluated by 2 researchers. If there are different opinions, we discuss them. If there are still differences exist, we would consult the third appraiser. Otherwise, we need to consult with the Cochrane Professional Group for solution.

#### 2.2.5 Assessment of heterogeneity

We will use a *x*^2^ test to estimate heterogeneity of both the MD and OR. Further analysis can be performed using the *I*^2^ test. If possible, we will also construct a forest plot for analysis. A random-effect model will be used to interpret the results if heterogeneity is statistically significant, whereas a fixed-effect model will be used if heterogeneity is not statistically significant. We will regard heterogeneity as substantial when *I*^2^ is greater than 50% or a low *P* value (<.10) is reported for the *x*^2^ test for heterogeneity.

#### 2.2.6 Sensitivity analysis

We will conduct a sensitivity analysis to identify whether the conclusions are robust in the review according to the following criteria: sample size, heterogeneity qualities, and statistical model (random-effects or fixed-effects model).

#### 2.2.7 Publication bias

If a result of a meta-analysis contains more than 10 articles and above, we will use a funnel plot to test the risk of publication bias.

#### 2.2.8 Quality of evidence

The quality of evidence for the main outcomes will also be assessed with the GRADE approach. The evaluation included bias risk; heterogeneity; indirectness; imprecision; publication bias. And each level of evidence will be made “very low,” “low,” erate,” or “high” judgment.

## 3 Discussion

As a chronic respiratory disease with a relatively high incidence in recent years, a large number of domestic and foreign scholars have conducted in-depth research on the etiology, clinical manifestations and treatment of asthma. Although asthma is currently a huge problem for medicine, the development of new drugs and new technologies has brought new expectations to asthma patients. Clinically, when selecting a treatment strategy, it is necessary to combine individual patient conditions and biomarkers to adopt personalized treatment.^[25]^

In recent years, the clinical RCT of asthma has been increasing, but it is still unsatisfactory in the diagnosis and treatment of diseases. Clinicians have not yet reached a consensus on the treatment principles and assessment of the disease, and lack uniform standardization standards. At present, there has not been a large-scale epidemiological investigation of the disease, and there are few reports in the literature. Chinese medicine has a profound theoretical foundation and rich clinical experience in the treatment of asthma. Acupuncture is an indispensable part of traditional Chinese medicine, with the characteristics of small side effects and easy operation. It has been used to treat a variety of systemic diseases such as diarrhea and cervical spondylosis.^[26]^ The therapy mainly regulates the balance of blood and blood by stimulating acupuncture points of the human body to achieve the effect of balancing yin and yang. The specific mechanism of acupuncture for asthma is still unclear, so we will use systematic reviews and meta-analyses to assess the efficacy and safety of acupuncture in the treatment of asthma.^[27]^ The results of this study may provide a possible ranking for acupuncture treatment of asthma.^[28]^ In addition, the scoring method will be used to assess the quality of the evidence for the primary outcome. We hope that these results will provide clinicians with the basis for acupuncture treatment of asthma and provide the best choice for patient care. In addition, although this study will conduct a comprehensive search, it will not search for languages other than Chinese and English, which will lead to some bias.

## Author contributions

**Data curation:** Yan Ming Chen, Sheng Deng

**Formal analysis:** Xiao-Lei Xie, Peng-Yun Xiao

**Funding acquisition:** Qiu-Hong Wang, Xu-Dong Yu

**Project administration:** Qiu-Hong Wang, Ji-Sheng Wang

**Supervision:** Peng-Yun Xiao, Yan Ming Chen

**Validation:** Xiao-Lei Xie, Peng-Yun Xiao

**Writing – original draft:** Yan Ming Chen, Xiao-Lei Xie

**Writing – review & editing:** Yan Ming Chen, Xiao-Lei Xie

## References

[1] Linde K Jobst K Panton J Acupuncture for chronic asthma. Cochrane Database Syst Rev 2000;2:CD000008. 10.1002/14651858.CD000008 10796465

[2] Martin J Donaldson ANA Villarroel R Efficacy of acupuncture in asthma: systematic review and meta-analysis of published data from 11 randomised controlled trials. Eur Respir J 2002;20:846. 12412674 10.1183/09031936.02.00078702

[3] Jobst KA Acupuncture in asthma and pulmonary disease: an analysis of efficacy and safety. J Altern Complement Med 1996;2:179–206. 9395653 10.1089/acm.1996.2.179

[4] Berger D Nolte D Acupuncture in bronchial asthma: bodyplethysmographic measurements of acute bronchospasmolytic effects. Am J Chin Med 1977;5:265–9. 10.1142/s0147291777000374 610977

[5] Sheng LL Yang HY Qin LF Effect of needling sensation reaching the site of disease on the results of acupuncture treatment of bronchial asthma. J Tradit Chin Med 1989;9:140–3. 2779278

[6] Brinkhaus B Acupuncture trial in patients with asthma – is a conclusion possible? Focus Altern Complement Ther 2010;13:274–5.

[7] Medici TC Acupuncture and bronchial asthma. Forschende Komplementarmedizin 1999;6: Suppl 1: 26–8. 10.1159/000057126 10077712

[8] Young-Qing Y Long-Ping C Shu-Lan M Anti-asthma effects of serum derived from adrenalectomized asthma rats treated with acupuncture (abstract). J Acupunct Tuina Sci 2005;3:5–15.

[9] Wang Y Yang YQ Ma SL SDS-PAGE analysis of components in serum with anti-asthma activity derived from rats treated by acupuncture. J Acupunct Tuina Sci 2009;7:8–12.

[10] Yang YQ Chen HP Wang Y Considerations for use of acupuncture as supplemental therapy for patients with allergic asthma. Clin Rev Allergy Immunol 2012;44:254–61. 10.1007/s12016-012-8321-3 22661215

[11] Kim IG Kim YI Hong KE The effects of Asari Herba Cum Radice (AHCR) herbal acupuncture at St36 on ovalbumin-induced asthma in C57BL mouse. Korean J Acupunct 2004;21:61–77.

[12] Ngai SPC Hui-Chan CWY Jones AYM A Short review of acupuncture and bronchial asthma — western and traditional Chinese medicine concepts. Hong Kong Physiother J 2006;24:28–38.

[13] Choudhury K Ffoulkescrabbe J Acupuncture for bronchial asthma. Altern Med 1989;155:206–1206.

[14] Tashkin DP Bresler DE Kroening RJ Comparison of real and simulated acupuncture and isoproterenol in methacholine-induced asthma. Ann Allergy 1977;39:379–87. 339785

[15] Jiang C Jiang L Qin Q Conventional treatments plus acupuncture for asthma in adults and adolescent: a systematic review and meta-analysis. Evid Based Complement Alternat Med 2019;2019:1–0. 10.1155/2019/9580670 PMC6354145 30792747

[16] Hassan N Shaaban H Ahmed Kamel I PO-1014 Effect of laser acupuncture on the immunomodulatory parameters in asthmatic children and its relation to asthma improvement. Arch Dis Child 2014;99: Suppl 2: A581.3-A581.

[17] Mohamed NH Shaaba HH Soliman MM The improvement in asthma severity and pulmonary functions after laser acupuncture application in asthmatic children. Med Res J 2014;13:93–9.

[18] Varon J Fromm RE Marik PE Acupuncture for asthma: fact or fiction? Chest 2002;121:1387. 12006413 10.1378/chest.121.5.1387

[19] Larsen MO Asthma and acupuncture. Ugeskrift Laeger 1988;150:2356. 3206612

[20] Li M Zhang X Bao H Acupuncture for asthma: protocol for a systematic review. Medicine 2017;96:e7296. 28658130 10.1097/MD.0000000000007296 PMC5500052

[21] Chu KA Wu YC Ting YM Acupuncture therapy results in immediate bronchodilating effect in asthma patients. J Chin Med Assoc 2007;70:265–8. 17631461 10.1016/S1726-4901(07)70002-3

[22] Davis PA Chang C Hackman RM Acupuncture in the treatment of asthma: a critical review. Allergol Immunopathol 1998;26:263–71. 9934404

[23] Jiao Y Wu Z Zhou W Explanation of evidence-based guidelines of clinical practice with acupuncture and moxibustion: adult bronchial asthma. Chin Acupunct Moxibustion 2016;36:529–31. 27509620

[24] Jun Z Suju S Zhong R Comparison between “five needles therapy” and conventional acupuncture for individual symptoms and signs of asthma of latent cold phlegm-fluid in the lung. Chin Acupunct Moxibustion 2018;38:7–11. 10.13703/j.0255-2930.2018.01.002 29354929

[25] Mehlmadrona L Augmentation of conventional medical management of moderately severe or severe asthma with acupuncture and guided imagery/meditation. Perm J 2008;12:9–14. 21339915 10.7812/tpp/07-110 PMC3037152

[26] Aldridge D Pietroni PC Clinical assessment of acupuncture in asthma therapy: discussion paper. J R Soc Med 1987;80:222–4. 3295235 10.1177/014107688708000411 PMC1290764

[27] Oĭvin VI Gaponiuk PI The combined use of traditional acupuncture and microacupuncture therapy in treating bronchial asthma patients. *Vopr Kurortol Fizioter Lech Fiz Kult*. 1989:24–27. 2756647

[28] Bahr F Bronchial asthma and auriculo-acupuncture (author's transl). Prax Klin Pneumol 1979;33: Suppl 1: 689–94. 461367

